# Ocular and Extraocular Expression of Opsins in the Rhopalium of *Tripedalia cystophora* (Cnidaria: Cubozoa)

**DOI:** 10.1371/journal.pone.0098870

**Published:** 2014-06-05

**Authors:** Jan Bielecki, Alexander K. Zaharoff, Nicole Y. Leung, Anders Garm, Todd H. Oakley

**Affiliations:** 1 Ecology, Evolution and Marine Biology, University of California at Santa Barbara, Santa Barbara, California, United States of America; 2 Marine Biological Section, University of Copenhagen, Copenhagen, Denmark; Lund University, Sweden

## Abstract

A growing body of work on the neuroethology of cubozoans is based largely on the capabilities of the photoreceptive tissues, and it is important to determine the molecular basis of their light sensitivity. The cubozoans rely on 24 special purpose eyes to extract specific information from a complex visual scene to guide their behavior in the habitat. The lens eyes are the most studied photoreceptive structures, and the phototransduction in the photoreceptor cells is based on light sensitive opsin molecules. Opsins are photosensitive transmembrane proteins associated with photoreceptors in eyes, and the amino acid sequence of the opsins determines the spectral properties of the photoreceptors. Here we show that two distinct opsins (*Tripedalia cystophora*-*l*ens eye *e*xpressed *o*psin and *Tripedalia cystophora*-*n*europil *e*xpressed *o*psin, or *Tc-leo* and *Tc-neo*) are expressed in the *Tripedalia cystophora* rhopalium. Quantitative PCR determined the level of expression of the two opsins, and we found *Tc-leo* to have a higher amount of expression than *Tc-neo*. *In situ* hybridization located *Tc-leo* expression in the retinal photoreceptors of the lens eyes where the opsin is involved in image formation. *Tc-neo* is expressed in a confined part of the neuropil and is probably involved in extraocular light sensation, presumably in relation to diurnal activity.

## Introduction

**Figure 1:**
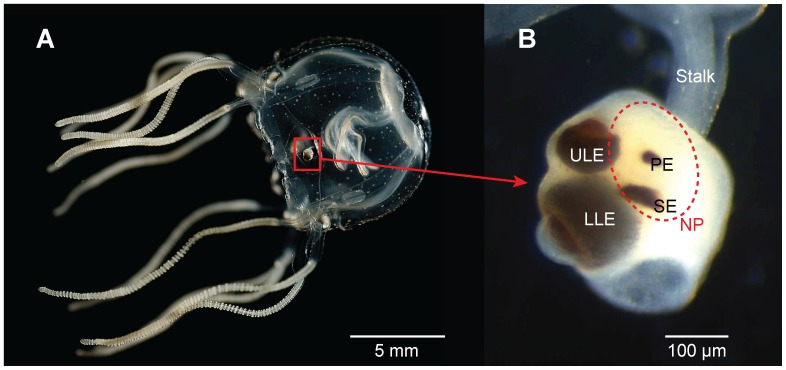
Cubozoan visual system. The visual system of the cubozoan *Tripedalia cystophora* (**A**) comprises four sensory structures called rhopalia (**B**). Each rhopalium carries six eyes of four morphological types (lower lens eye LLE, upper lens eye ULE, pit eye PE and slit eye SE) and a light sensitive neuropil (NP, red broken line). The eyes are responsible for the image formation in the animal and the light sensitive neuropil is thought to be involved in diurnal activity.

Cubozoans are an emerging model system for understanding visual information processing through integrative studies of morphology, behavior and physiology. Morphologically, cubozoans accomplish image analysis with a limited, and therefore experimentally tractable, neural capacity of about one thousand neurons [1], [2]. Coupled with this simple neural architecture is a complex visual system of 24 eyes: Cubozoans have six eyes on each of four sensory structures called rhopalia. Two eyes per rhopalium are lens eyes, comparable in morphology to vertebrate eyes (Figure 1). Behaviorally, cubozoans use vision to avoid obstacles [3]–[5], to navigate using terrestrial cues [6] and for phototaxis [7], [8]. Multiple behaviors of cubozoan medusae are modulated by a swim pacemaker system that is influenced by light sensed by the lens eyes, pit eyes, and the neuropil [9], [10]. Physiologically, the cubozoan *Tripedalia cystophora* possesses monochromatic vision with peak sensitivity in the blue-green part of the spectrum (504 and 512 nm for the upper and lower lens eyes respectively) [11], [12]. Since the specific peak absorbance values were obtained by electroretinograms (ERGs), the difference is insufficient to clearly indicate distinctive maxima between the eyes.

In addition to morphology, behavior and physiology, scientists are beginning to learn the genetic basis of visual information processing in cubozoans. Like most other animals, cubozoans use light sensitive transmembrane proteins called opsins [13]–[16]. Using immunohistochemical staining and *in situ* hybridization, Koyanagi and colleagues [17] reported one opsin from the cubozoan *Carybdea rastonii* with expression in the upper and lower lens eyes. Herein, we refer to this gene as *Cr-leo* (*Carybdea rastonii l*ens eye *e*xpressed *o*psin). Koyanagi et al. expressed *Cr-leo* heterologously and found the purified pigment to have an absorption maximum of about 500 nm, in close concordance with ERG results from lens eyes of *T. cystophora* [11]. Another cubozoan opsin is also known: Kozmik and colleagues [18] reported an opsin in *T. cystophora*. They also reported expression in both lens eyes using immunohistochemical staining. Unlike *Cr-leo*, the *T. cystophora* opsin showed with heterologous expression maximum absorption to blue light (∼470 nm), quite different from the ERG maximum of 504 to 512 nm reported for the same species. Furthermore, the primary amino acid sequences of opsins from *C. rastonii* and *T. cystophora* are different enough to indicate they might be paralogs and not orthologous.

In addition to opsins expressed in animal eyes, many other opsins have extraocular expression, with functions such as circadian entrainment, pupil response, and nematocyte modulation. Although direct molecular evidence for extraocular opsin expression is unknown in Cubozoa, previous researchers suggested the neuropil might express opsins because it influences the swim pacemaker in different light conditions [10]. In Cnidaria beside cubozoans, there is direct molecular evidence for extraocular opsin expression [14], [19]. In the hydrozoans *Cladonema radiatum* and *Podocoryne carnea*, several opsins are expressed in tissues that lack obvious visual functions, including tentacles, gonads, and manubrium [20]. In *Hydra magnipapillata*, many different opsin-like sequences exist [20], [21] and some are expressed broadly in neurons dotting the ectoderm [15], [22]. *Hydra magnipapillata* has no eyes, and they use extraocular phototransduction in dermal photoreception [22] and to modulate firing of nematocytes in different light levels [23].

Here, we report that the rhopalia of *T. cystophora* express at least two opsins; one identical to that found previously by Kozmik [18] and one closely related to *Cr-leo* from *C. rastonii* [17]. Based on quantitative PCR we found the two opsins to be expressed at significantly different levels in the rhopalium. Additionally, *in situ* hybridization localized the expression of the two opsins to different rhopalial structures, suggesting that one opsin serves an extraocular function. We find the opsin transcript found previously by Kozmik to be expressed in the neuropil of *T. cystophora*, but we see no evidence of expression of that transcript in the lens eyes. We refer to this gene as *Tc-neo* (*T. cystophora n*europil *e*xpressed *o*psin). Using phylogenetic analyses, we show the novel *T. cystophora* gene to be orthologous to *Cr-leo* and transcripts of this gene are expressed in the lens eyes, leading to the acronym *Tc-leo*. Because cubozoans are valuable organisms for gaining an integrative understanding of visual information processing, learning about opsin expression and function is critical. Our expression results corroborate earlier hypotheses for a light sensing function for the neuropil, perhaps mediated through opsin, which may have a different maximal wavelength sensitivity compared to the lens eye opsin.

## Methods

### Animals

We collected medusae of *Tripedalia cystophora*, Conant 1897, near La Parguera, Puerto Rico (N17° 58′ 22.48″ W67° 04′ 03.66″), in the mangrove where they feed on copepods aggregated in light shafts. *T. cystophora* is not an endangered or protected species and specific permissions were not required to collect the animals. We stored the medusae in RNAlater for transcriptome sequencing. In addition to the collected animals, we obtained medusae (7–9 mm in bell diameter) of *T. cystophora* from our cultures at the University of Copenhagen, Denmark. Cultures originated from gravid females collected from La Parguera, Puerto Rico. In the cultures the medusae are kept in a 250 l tank with circulating seawater at 30‰ and about 28°C and fed SELCO-enriched (INVE Technologies, Dendermonde, Belgium) *Artemia* daily. They reach adult size in 2–3 months.

### 454 Pyrosequencing

We constructed cDNA from approximately 25 rhopalia dissected from *T. cystophora*. We extracted RNA using the Nucleospin RNA XS isolation kit (Macherey-Nagal, Bethlehem, PA, USA). Purified RNA was quantified on a Qubit Fluorometer (Invitrogen, Grand Island, NY, USA). We generated cDNA using the SMARTer cDNA synthesis kit (Clontech, Mountain View, CA, USA). To reduce sequencing artifacts due to poly-T tracts, we used a modified 3′ primer for first strand synthesis: 454 poly-T (please refer to Table 1 for primer sequences). We conducted second strand synthesis using the amplification protocol outlined in the SMARTer cDNA kits, with a cycle number of 25. Amplified cDNA was purified using a standard phenol:chloroform:isoamyl protocol and quantified on a Qubit fluorometer (Invitrogen, Grand Island, NY, USA). We pooled separate second strand reactions to reach a concentration of 3.44 µg for the cDNA pool. The resulting cDNA samples were shipped to Brigham Young University for titanium pyrosequencing using the Roche 454 platform, according to manufacturer’s instructions, employing partial runs with barcodes. We assembled the new transcriptome data with GS De Novo Assembler v2.3 (‘newbler’; 454 Life Sciences/Roche, Indianapolis, IN, USA) to create a cDNA de novo assembly with default threshold options. We used LUCY [24] to trim low quality nucleotide reads and deleted any assembled contigs below 100 nucleotides in length. We found sequences similar to opsin using BLASTP similarity searches [25]. We deposited raw reads from our transcriptome in the NCBI SRA database (SRR1182852).

**Table 1:** PCR Primer sequences used for various molecular techniques.

Primer name	Primer sequence 5′→3′
454 poly-T	AAG CAG TGG TAT CAA CGC AGA GTA CTTTTTT CTTTTTT
RT-PCR LEO-F	CTG GAA GGT GCG ATA GCA TT
RT-PCR LEO-R	AGG TTG CCG CCT TCT TTA TT
RT-PCR NEO-F	CGC TGG AAG CGC CTG TTG CAG
RT-PCR NEO-R	TCA TTC CGG CTC AAC AGA ATT TCC
qPCR LEO-F	GGC CTT TCG TCG CAA CCG CT
qPCR LEO-R	CGG CCA GTT GAT GGA GCA TCG C
qPCR NEO-F	CGC TGG AAG CGC CTG TTG CAG
qPCR NEO-R	TGG TGT CCC GCT TCA AGG GAA GT

Primer sequences used for the various molecular techniques: 454- pyrosequencing, reverse transcriptase PCR (RT-PCR) and quantitative PCR (qPCR). F and R denote forward and reverse primers respectively.

### Reverse Transcriptase PCR

To confirm expression of opsin genes found in transcriptome sequence data, we performed Reverse Transcriptase PCR (RT-PCR). We first prepared cDNA from approximately 10 whole rhopalia by Trizol extraction of mRNA followed by reverse transcription into cDNA. We then performed RT-PCR amplification using opsin specific primers based on sequences obtained from the 454-pyrosequencing data (*Tc-leo*) and from previously published data (*Tc-neo*) [18]: RT-PCR *Tc-leo*-F & R and RT-PCR *Tc-neo*-F & R (Table 1). Amplification conditions: RT step - 10 min at 50°C, Inactivation step - 5 min at 95°C, Cycle 35X - 10 sec at 95°C and 15 sec at 55°C. We deposited our sequence of *Tc-leo* in GenBank (accession no. KJ542646).

### Quantitative PCR (qPCR)

**Figure 2:**
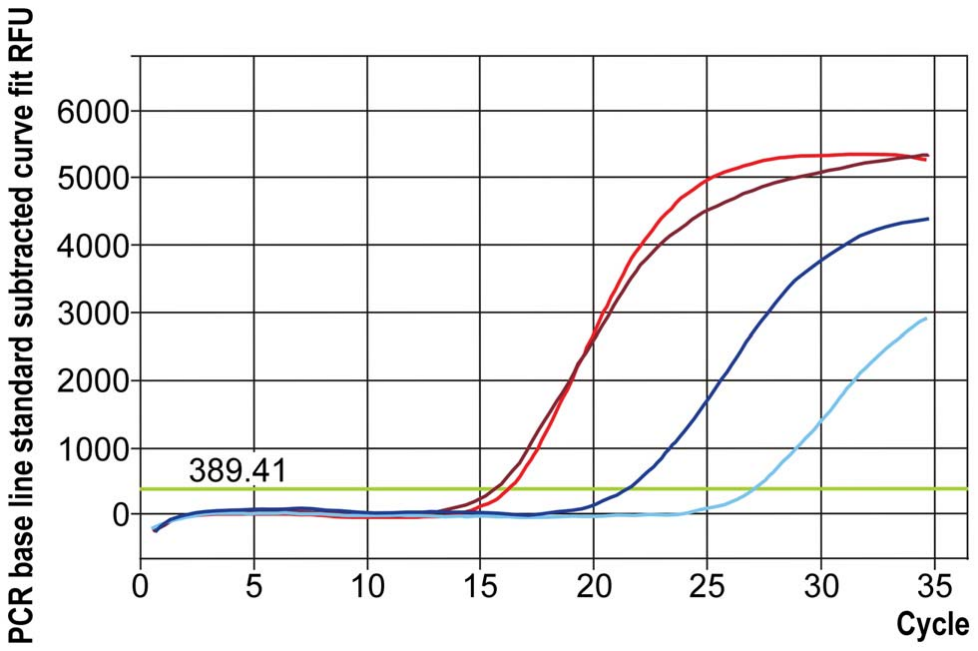
Graphical representation of the quantitative amplification of *leo* and *neo*. Example of a qPCR amplification run of *Tc-leo* and *Tc-neo*, showing the relative relationship between *Tc-leo* and *Tc-neo* expression in the *Tripedalia cystophora* rhopalium. It is evident that *Tc-leo* is expressed at higher levels than *Tc-neo* (see also Table 2). The two red shades each represent one replicate of *Tc-leo* and the two blue shades correspond to *Tc-neo* replicates. Green horizontal line depicts the threshold value, and the threshold cycle (C_T_) is determined by the cycle number, at which the concentration exceeds the threshold. The lowest C_T_ value for a replicate was chosen for each opsin in each run. Eight runs were performed.

We used quantitative PCR to test the hypothesis that *Tc-leo* and *Tc-neo* are expressed at different levels in the rhopalium. We constructed cDNA as for reverse transcriptase PCR, except using only one rhopalium for each run. We performed qPCR using eight biological replicates (8 separate rhopalia from 8 different animals) with two to four technical replicates each to compare relative expression of *Tc-leo* and *Tc-neo* in the rhopalial transcriptome. In seven replicates we were able to include a sham (no RNA) negative control. We designed qPCR primers to each opsin. No introns are known from these genes, so we relied on DNAse treatment to prevent DNA contamination. Primers used were qPCR *Tc-leo*-F & R qPCR *Tc-neo*-F & R (Table 1). Amplification conditions: RT step - 10 min at 50°C, Inactivation step - 5 min at 95°C, Cycle 40X - 10 sec at 95°C and 15 sec at 55°C, Melt Curve Analysis 80X - 30 sec every 0.5°C. We used the iScript One-Step RT-PCR Kit with SYBR Green (BioRad, Hercules, CA, USA) for all quantitative PCR runs (Figure 2 and Table 2).

**Table 2:** Relative expression of the rhopalial opsins based on quantitative PCR.

Biological Replicate	*Tc-leo* Average C_T_±SEM	*Tc-neo* Average C_T_±SEM
1	15.86±0.30	25.05±1.91
2	19.55±2.01	28.30±1.45
3	20.55±0.05	32.50±0.00
4	26.40±4.53	No C_t_ Value Given
5	20.07±0.38	No C_t_ Value Given
6	33.35±0.15	No C_t_ Value Given
7	34.23±1.01	No C_t_ Value Given
8	29.70±1.85	31.5±0.46

Quantitative PCR ascertain the relative expression of *Tc-leo* and *Tc-neo*. In every run of the qPCR *Tc-leo* expressed relatively higher than *Tc-neo*. In every run the cycle threshold (C_T_) was lower for *Tc-leo*. In four runs *Tc-neo* was not sufficiently expressed to return a C_T_ ascertaining the much higher relative expression of *Tc-leo.*

### *In situ* Hybridization

**Figure 3:**
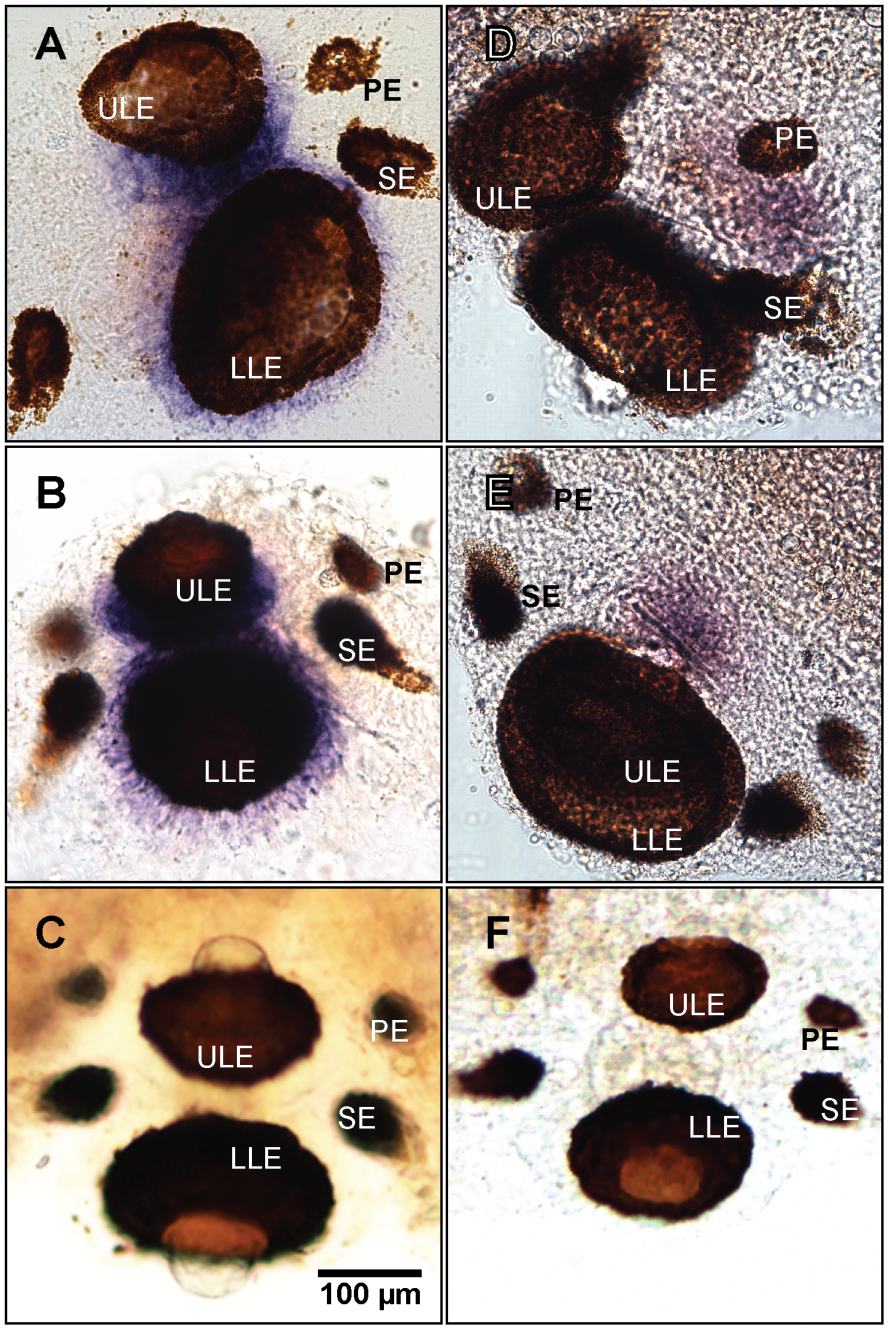
Opsin expression in the rhopalium of *Tripedalia cystophora*. *In situ* hybridization colorimetric staining places *Tc-leo* mRNA expression in the cell bodies of the retinal photoreceptors of the lens eyes (upper lens eye ULE and lower lens eye LLE) (**A,B**). The control with the sense probe (**C**) is devoid of colorimetric staining validating the positive results in **A** and **B**. *Tc-neo* mRNA is expressed in part of the neuropil (**D,E**), which is also known to have photosensitive properties [10]. *Tc-neo* sense control is seen in **F**. None of the opsins are expressed in the lesser eyes (pit eyes PE and slit eyes SE), suggesting that other opsins could be expressed in these eye types.

**Figure 4:**
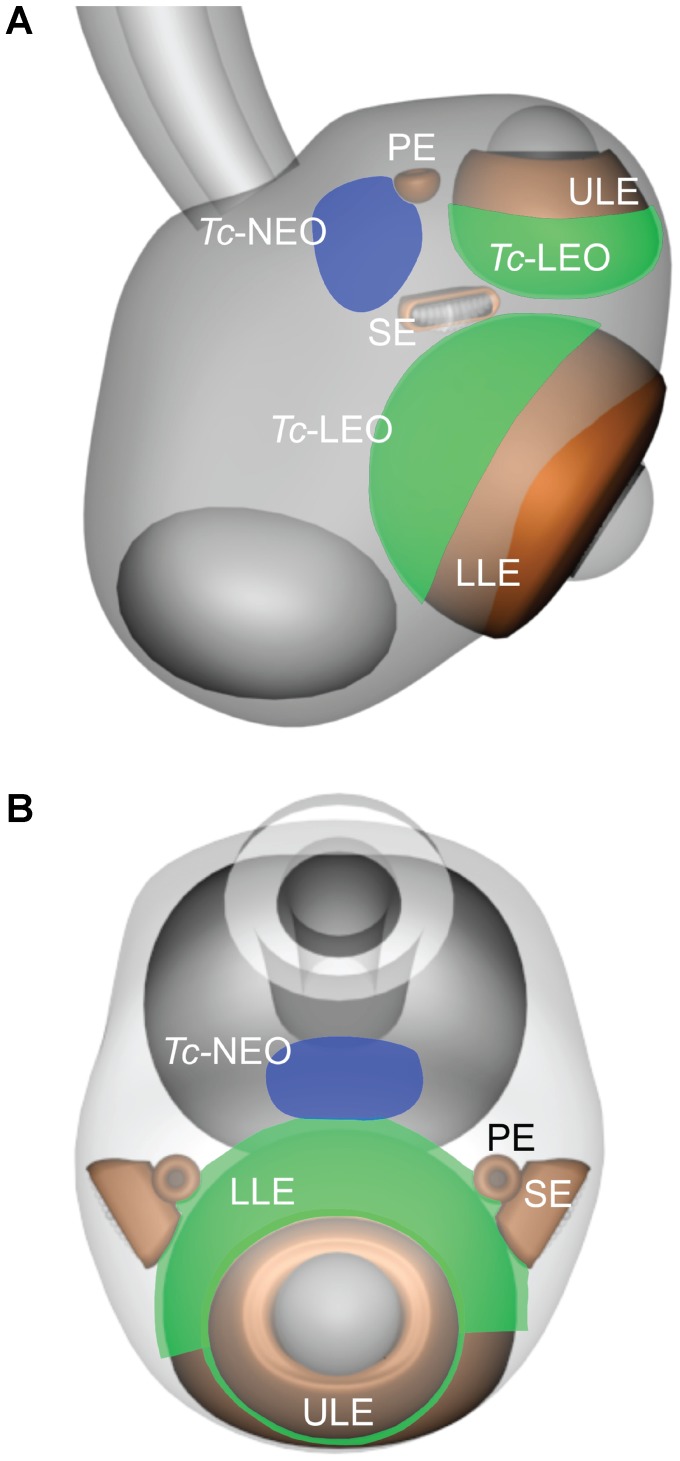
Graphical representation of expression of *Tc-leo* and *Tc-neo*. While the *Tc-leo* is expressed in the retinal photoreceptors of the lens eyes, *Tc-neo* is expressed in the neuropil. The green areas depict the rhopalial *in situ* hybridization colorimetric staining pattern of the *Tc-leo* and blue areas represent *Tc-neo* (**A**, side view and **B**, top view). Upper lens eye (ULE), lower lens eye (LLE), slit eye (SE) and pit eye (PE).

For the *in situ* hybridization experiments we fixed cultured medusa for 24 hrs in 4% PFA, rinsed 3×5 mins in PBS and dehydrated in a graded series of methanol (25, 50, 75, 90 and 100%). The experiments followed the colorimetric labeling protocol established by Grens et al. [26] with modifications by Plachetzki et al. [22]. To generate *in situ* hybridization probes, we used RT-PCR to amplify portions of each opsin, using the same primers described above (qPCR *Tc-leo* and qPCR *Tc-neo*). In addition to experimental anti-sense probes, we generated a sense control for both the opsins to verify the validity of the peroxidase staining. The rhopalia were whole mounted and the opsin expression was recorded by digital imaging (Figure 3). *Tc-leo* and *Tc-neo* expression was graphically illustrated (Figure 4).

### Phylogenetic Analyses

**Figure 5:**
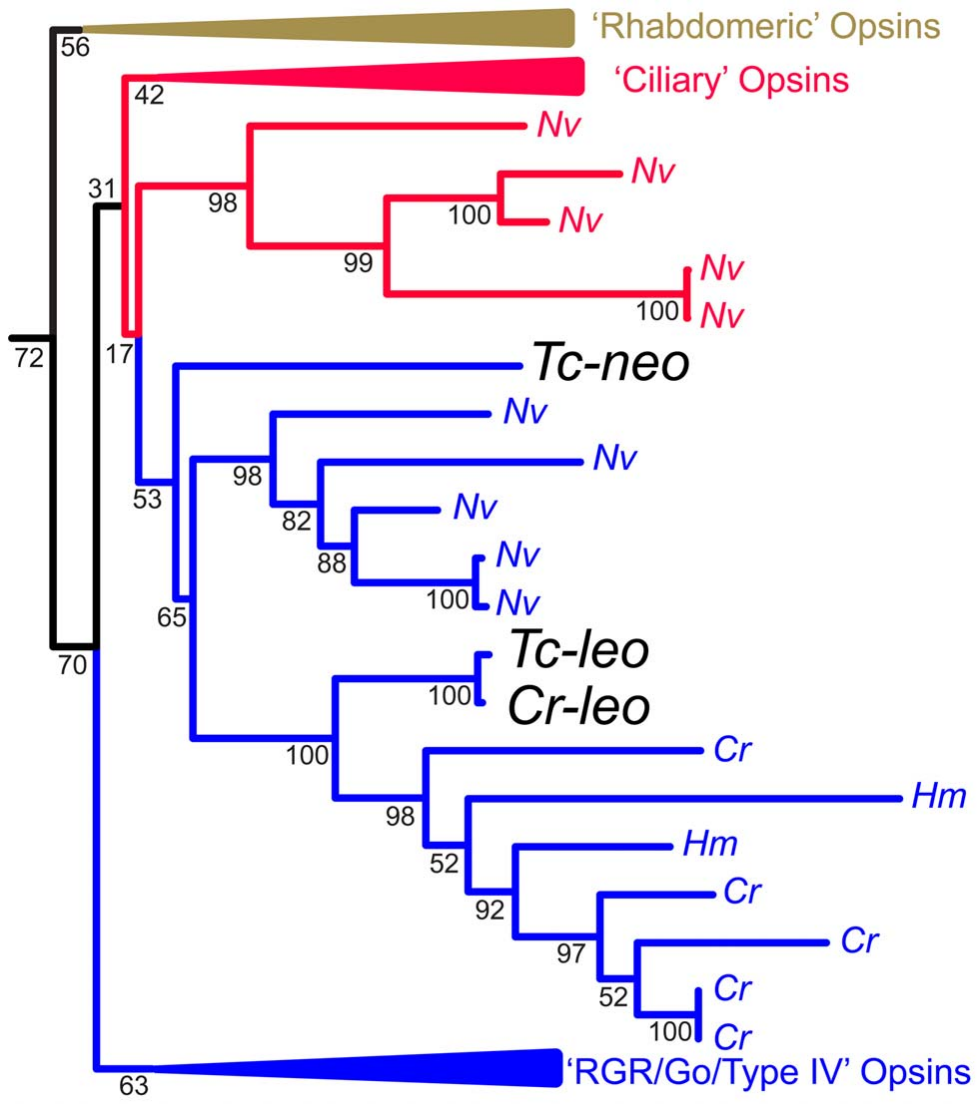
Cnidops phylogenetic tree. Maximum likelihood phylogenetic analysis including representative animal opsins from the “O&O” data set of Feuda et al. [27] plus additional Cnidarian opsins indicates that *Tc-leo* and *Tc-neo* are distantly related opsins whereas *Tc-leo* and *Cr-leo* are closely related to each other. Illustrated here is a subset of all the genes analyzed, focusing on Cnidarian opsins; *Cladonema radiatum* (*Cr*), *Hydra magnipillata* (*Hm*) and *Nematostella vectensis* (*Nv*). The colors of the branches correspond to their phylogenetic placement in the analysis of Feuda et al. [27]. The full phylogeny, showing all genes analyzed is included in Figure S2. Numbers at nodes are bootstrap values based on 100 pseudoreplicated datasets, implemented in RAxML [29], assuming a GTR plus gamma model of protein evolution.

We explored the phylogenetic relationship of the three known cubozoan opsins (Figure 5). We used the “O&O” opsin data set of Feuda et al. [27], which contains 104 sequences that are representative of all major opsin subfamilies, plus placopsin and melatonin receptor outgroups. This data set already included *Cr-leo*, and we added both *Tripedalia* opsins, *Tc-leo* and *Tc-neo*, by using the ‘-add’ option of MAFFT 7.0 [28] to align those two protein sequences to the published alignment of Feuda et al. [27]. We searched for the Maximum Likelihood gene tree, assuming a GTR+gamma model of protein evolution, using RAxML [29]. We also gauged node support using 100 bootstrap pseudoreplicates, also implemented in RAxML. We performed all phylogenetic analyses in Osiris, within the Galaxy bioinformatics package [30], [31]. All data and analyses are publicly available (http://galaxy-dev.cnsi.ucsb.edu/osiris/u/ostratodd/p/leo-neo).

### Light Microscopy and TEM

**Figure 6:**
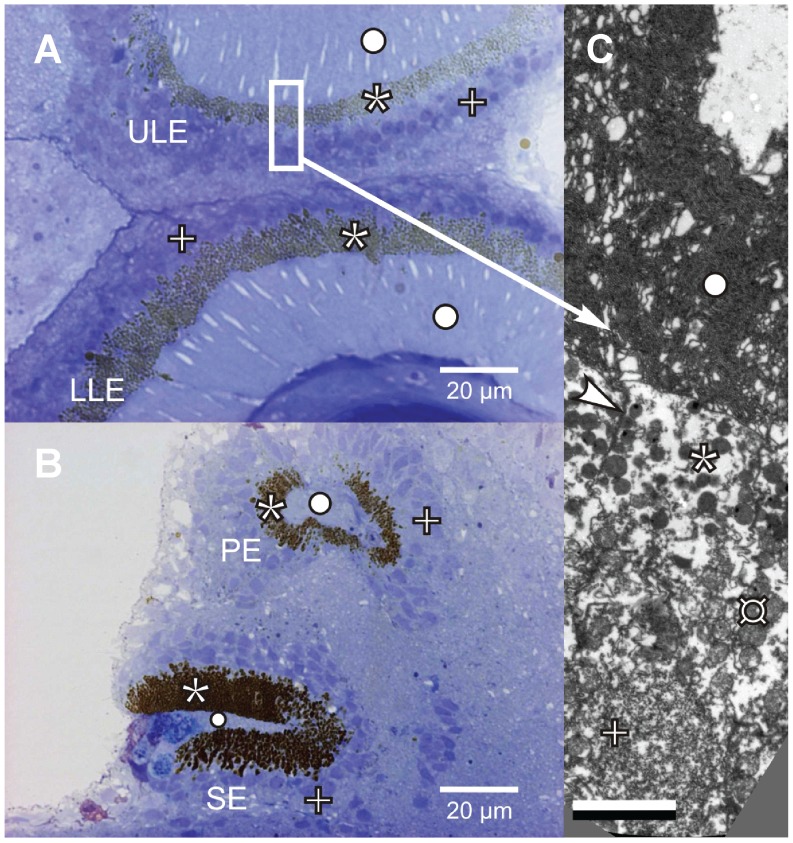
Microscopy of the eyes of *Tripedalia cystophora*. Light microscopy of the upper and lower lens eyes (**A**) and pit and slit eyes (**B**) show that the nuclei (+) are located in the cell bodies outside the zone of pigment granules (*). Transmission electron micrograph (**C**) shows the cell membrane (arrowhead) and numerous mitochondria (¤) that are located between the pigment granules and the nucleus suggesting the area of protein translation to be adjacent to the nucleus. The folded membranes of the cilium (○) are evident in the outer segments of the photoreceptor cells (C). Scale bar in (**C**) 2 µm.

To confirm the position of the opsin expression in the rhopalium, we made light microscopy and ultrastructural images from rhopalia prepared by standard EPON embedding and sectioning procedures (see [1]) (Figure 6).

## Results

### 454 Pyrosequencing

Our search of resulting transcriptome data returned a previously unknown *Tripedalia cystophora* opsin sequence that is 94% identical to *Cr-leo*, the lens eye opsin described from *Carybdea rastonii* [17]. We did not find any other opsin sequences in the rhopalial 454 data, but this is probably due to the limited depth of coverage of our transcriptome, which may not detect sequences with low levels of expression.

### Reverse Transcriptase PCR (RT-PCR)

We used reverse transcriptase PCR to verify expression in rhopalia of the previously published *Tc-neo* [18] and our newly found *Tc-leo*. We found both opsins to be expressed in cDNA prepared from rhopalia. However, a difference in band brightness on the gel suggested that *Tc-leo* was expressed at a higher level than *Tc-neo* (data not shown).

### Quantitative PCR (qPCR)

Our qPCR results confirmed the differences in the level of expression of the two opsins in *T. cystophora* rhopalia. The level of expression of *Tc-leo* was higher than *Tc-neo*. In all 8 biological replicates *Tc-leo* returned a lower cycle threshold (C_T_) than *Tc-neo* (Table 2). This result is significant in a binomial test (p = 0.0078). In four replicates the *Tc-neo* expression was so low that it did not return a C_T_ value, whereas *Tc-leo* was expressed in all replicates. These results clearly and conservatively show that *Tc-leo* has significantly higher expression than *Tc-neo* in the rhopalia of *T. cystophora*.

### *In situ* Hybridization

The colorimetric *in situ* hybridization determined the location of *Tc-leo* and *Tc-neo* mRNA expression (Figures 3 and 4). *Tc-leo* mRNA was expressed in the cell bodies of the photoreceptors of the upper and lower lens eyes (Figures 3A, B and 6). The photoreceptors of cubozoans include an outer photoreceptive segment, a mid-section containing pigment granules and a basal cell body containing the nucleus (Figure 6). The lens eyes have everted retinas and the nuclei of the photoreceptors are located outside the pigment screen (Figures 6 and S1) [32]. The colorimetric labeling using our *Tc-leo* probe is located in the area of the photoreceptors corresponding to the nucleus and thereby the endoplasmatic reticulum. *Tc-leo* expression is limited to the retinal photoreceptors of the lens eyes; none of the surrounding tissue is stained and the sense control is devoid of colorimetric staining (Figure 3C). In contrast to *Tc-leo*, *Tc-neo* was expressed in the neuropil of the rhopalium (Figure 3D and E). The neuropil fills up most of the volume of the rhopalium between the epidermis and the gastrodermis, from the base of the stalk to the top of the lower lens eye [2]. *Tc-neo* was not expressed in the entire neuropil but in limited parts (Figure 3D and E). Curiously, we did not find evidence that either opsin is expressed in photoreceptors of the pit and slit eyes of the rhopalium (Figure 3).

### Phylogenetic Analysis

We found (Figure 5) *Tc-leo*, *Cr-leo* and *Tc-neo* to fall into a clade with other cnidarian opsins, although with fairly low bootstrap support of 53%. This opsin clade contains genes from the hydrozoans *Hydra magnipapillata* and *Cladonema radiatum* and from the anthozoan *Nematostella vectensis*. This cnidarian clade of opsins may be called ‘cnidops’ [15]. In the analysis of Feuda et al. [27], cnidops is the sister-group of the bilaterian RGR/Go clade (or type IV opsins sensu [16]) of opsins. However, when we aligned *Tc-leo* and *Tc-neo* to the alignment of Feuda et al. [27], we did not recover this relationship. Instead, we find a group of opsins from *Nematostella* that Feuda et al. [27] found to be related to ciliary opsins to be the sister group of cnidops with very low support (17%). These results indicate that opsin phylogenetic results, especially for ancient nodes, are highly sensitive to which genes are included.

Within the cnidops clade, we found *Tc-leo* and *Cr-leo* to be well-supported (100% bootstrap) orthologs. These two orthologs form a sister group to multiple hydrozoan opsins from *H. magnipapillata* and *C. radiatum*. In contrast, *Tc-neo* is much more distantly related, and not placed with certainty in our analysis. Our results do not recover a close relationship between c-opsins and *Tc-neo* (for detailed phylogenetic analysis, please refer to Figure S2).

## Discussion

With a relatively simple nervous system coupled with camera-type, image-forming eyes, cubozoans have great potential to become a model system to understand visual information processing. In addition to numerous publications about morphology, physiology and behavior, research into the molecular basis of cubozoan light sensitivity has also begun [17], [18], [33]. Here, we report expression of two different opsins in the cubozoan *Tripedalia cystophora*. We find *Tc-leo* to be expressed in the upper and lower lens eyes and *Tc-neo* to have extra-ocular expression in the neuropil. Even though previous techniques only found one opsin per species, our phylogenetic results indicate the duplication of *leo* and *neo* occurred before the origin of cubozoans. These results reconcile previous discrepancies between molecular and physiological data and provide the first direct molecular evidence of extraocular opsin expression in a cubozoan.

### Leo Expression in Lens Eyes

Our results indicate *leo* mRNA is expressed in the cell body of the lens eye photoreceptors, corresponding to the area of the cell nucleus (Figures 3, 6 and S1). Light microscopy and ultrastructural studies confirm the position of the nuclei in the cell bodies (Figure 6) [32], [34]. Our *leo* expression results are consistent with Koyanagi et al. [17], but different from the results of Kozmik et al. [18] who reported a different gene (*Tc-neo*) to be expressed in lens eyes with a maximal sensitivity to blue light (470 nm) in *in vitro* expression analysis. In contrast to the 470 nm peak of *Tc-neo*, ERG experiments found a single sensitivity peak near 510 nm in lens eyes of the same species [11]. We find *Tc-leo* to be very similar and orthologous to an opsin (*Cr-leo*) of a related cubozoan and *Cr-leo* has an *in vitro* absorption maximum of 500 nm [17], much more closely matching the physiological results of *T. cystophora* lens eyes. These results can be reconciled if only *leo* (not *neo*) is expressed in lens eyes. One possible explanation for the discordant result is that Kozmik et al. [18] obtained non-specific antibody staining in the lens eyes, such that their antibody probed *Tc-leo* rather than *Tc-neo*. To generate their *Tc-neo* antibody, they used the c-terminal 55 amino acids of the opsin. In this region, we found that 30% of the amino acids are identical and 63% of amino acids have similar physicochemical properties between *Tc-leo* and *Tc-neo*. Because *in situ* hybridization used sequence-specific probes, and immunohistochemistry may be more prone to cross-hybridization, we suggest non-specific hybridization as the cause of the discordant expression results.

### Neo Expression in Neuropil

In addition to *Tc-leo* expression in lens eyes, we find *Tc-neo* to be expressed in the neuropil and to have a lower level of overall expression in rhopalia. These results and previous research suggest functional involvements of *neo* in the rhopalium, and we therefore hypothesize the neuropil to contain an aggregated/higher-order extraocular photoreceptor (sensu [19]). This is further supported by electrophysiological data where the neuropil was shown to modulate the pacemaker signal frequency when exposed to light [10]. *Tc-neo* has a peak absorbance of ∼470 nm [18] consistent with other photosensitive pigments involved in diurnal activity pattern (entrainment), which often have absorption maxima in the blue spectrum of visible light [35], [36]. *T. cystophora* display light mediated diurnal behavior [37] and since *Tc-neo* is expressed in the neuropil, which is transparent and exposed to ambient light, it is possible that this opsin is involved in the overall activity pattern of the animal based on the ambient light level [37]. The results on level of expression also fit well with receptor morphology where the membranes of cells with non-directional photoreception usually are considerably less folded than retinal photoreceptors used for spatial vision [38], and extraocular opsins should have lower expression than ocular opsins. From the qPCR experiments it is evident that *Tc-neo* is expressed around 200 times less than *Tc-leo*. This lower degree of expression of *Tc-neo* compared to *Tc-leo* is also evident when comparing the strength of colorimetric staining. Despite multiple lines of evidence suggesting *Tc-neo* involvement in neuropil-based light sensitivity functions, we caution that firm conclusions await direct experimental manipulation of *Tc-neo*, which await the advent of genetic manipulation techniques in cubozoans.

### Leo and Neo are Distantly Related Opsins

The two opsins we found expressed in the rhopalium of *T. cystophora* are rather distantly related, yet both appear to be members of a cnidarian opsin clade that can be called cnidops [15]. The close relationship of one of these genes (*Tc-leo*) to another gene (*Cr-leo*) expressed in lens eyes, suggests that all cubozoan lens eyes express a member of this orthologous opsin clade. Our conclusion placing the second opsin (*Tc-neo*) within cnidops is different from a previous conclusion that *Tc-neo* is a c-opsin [18]. Despite different conclusions, the results are not drastically different. Kozmik et al. [18] found *Tc-neo* to be sister to c-opsins with low (49%) bootstrap support. Support for *Tc-neo* placement on our tree was similarly low (53%), so definitive conclusions await further data. One way forward to placing this opsin with greater certainty would be to include additional orthologs. While we did not find clear orthologs in the genomes of *Nematostella* or *Hydra*, other cubozoans besides *Tripedalia* and *Carybdea* may have these genes, which could break up the long-branch leading the *Tc-neo* allowing more reliable placement.

The distant relationship between *leo* and *neo* indicate the possibility of functional differences in the signal transduction pathway of *leo* and *neo*. We know from thorough work by Koyanagi and colleagues [17] that *leo* initiates signaling through a Gα_s_ signal transduction pathway. They used a combination of *in vivo* and *in vitro* experiments to show that *Cr-leo*, Gα_s_, and adenylyl cyclase act to alter cAMP levels during phototransduction. In contrast to *leo*, we do not have firm knowledge of the signal transduction pathway of *neo*. Although Kozmik et al. [18] proposed that *Tc-neo* relies on a Gα_i/t_ pathway based on EST expression in rhopalia of guanylate cyclase and phosphodiesterase, no functional experiments are yet published for these genes. Since these genes are involved in numerous processes besides phototransduction, cubozoans could use these genes in other ways. Therefore, the presence of two distantly related and functionally different phototransduction pathways in cubozoans is possible, but definitive conclusions await further research.

### Additional Rhopalial Opsins?

Based on their phylogenetic position, we hypothesize that genes homologous to both *leo* and *neo* are present in all cubozoans, and based on the totality of expression studies conducted thus far, they are expressed in lens eyes and neuropil, respectively. Yet two previous studies and ours only found one opsin per species, using three different methods. Koyanagi and colleagues [17] used degenerate primers, Kozmik generated an expressed sequence (EST) cDNA library from rhopalial mRNA [18], and we used 454-pyrosequencing of cDNA. We confirmed expression of the previously discovered *Tc-neo* with PCR and *in situ* hybridization, but never found this gene in our 454 sequence data. These results provide a cautionary tale, illustrating that absence of evidence is not evidence of absence. In fact, the lack of known opsin expression associated with the pit and slit eyes likely indicates that there still may be undiscovered opsins in the rhopalium.

Even though the photoreceptor nuclei of the pit and slit eyes are also located in the cell bodies outside the pigment granules (Figure 6B), there is no evidence of *Tc-leo* or *Tc-neo* expression in the pit or slit eyes of the rhopalium. This result is curious since most animal eyes utilize opsins as their light sensitive molecule. One possible exception to an opsin-based eye is the pigment ring eye of the sponge *Amphimedon queenslandica*: Opsin is absent from the species’ genome and a Cryptochrome (Cry) transcript is expressed at the pigment ring larval eye, although functional experiments have not yet confirmed that Cry mediates phototaxis [39]. One possible explanation is that these small eyes utilize one or more opsins that are expressed at such low levels that we missed them in the 454-pyrosequencing techniques. The paucity of information on pit and slit eyes leaves open the possibility that they have different spectral properties than the lens eyes. A system with overlapping visual fields (slit eye and lower lens eye) but different spectral sensitivity could be used in depth assessment, since the spectral composition varies predictably with depth. Comparing the relative response of the two eyes could then provide information of the vertical placement of the animal in the water column [38].

## Summary

From our combined qPCR and *in situ* hybridization results there is little doubt that the *Tc-leo* is expressed in the retinal photoreceptors of the lens eyes and is as such the first step in image formation. *Tc-neo* is expressed in the neuropil and highly likely involved in extraocular light sensation, presumably in relation to control the diurnal activity pattern [37].

## Supporting Information

Figure S1 **Graphical representation of cubozoan photoreceptor morphology.** Sagittal section of the lower lens eye of *Tripedalia cystophora* (**A**) (modified from [34]). Light is absorbed in the ciliary layer by the photoreceptive outer segments (POS) (**B**) and the pigment layer prevents false light entering the eye. Pigment granules (pg) make up the pigment layer and are located in the pigmented region (PR) of the photoreceptors. The nuclei (n) are located in the nuclear region (NR) of the cell bodies. The photoreceptors are everted and the neural layer is thereby located outside the pigment layer. Gray et al. [40] found invaginated synapses (is) in the nuclear region but the significance of this discovery is largely unknown. It is thought that the photoreceptors articulate on second order neurons since the proximal end protrudes into a neural plexus that extends into the neuropil of the rhopalium.
(TIF)

Figure S2 **Detailed opsin phylogenetic tree.** Maximum likelihood phylogenetic analysis including representative animal opsins from the “O&O” data set of Feuda et al. [27], plus the new *Tc-leo* gene. Feuda et al. [27] did not include *Tc-neo*, which we also added to their data set. We rooted animal opsins with melatonin receptor genes (black branches). The branch colors for animal opsins follow Feuda et al. [27]. Unlike Feuda et al. [27], we do not recover monophyletic ciliary opsins (red branches). Also differing from Feuda et al. [27], we do not find a sister-group relationship between ‘cnidops’ [41] genes and the clade called Type IV opsins by Porter et al. [16]. The difference between our topology and that of Feuda et al. [27] seems to be caused by the addition of *Tc-neo*. Numbers at nodes are bootstrap values based on 100 pseudoreplicated datasets, implemented in RAxML [29], assuming a GTR plus gamma model of protein evolution, the same model used by Feuda et al. [27].
(TIF)
