# Supplementary figures and images for: Ocular and Extraocular Expression of Opsins in the Rhopalium of Tripedalia cystophora (Cnidaria: Cubozoa)
Source: PLoS One. 2014 Jun 5;9(6):e98870. doi: 10.1371/journal.pone.0098870 (PMC4047050; doi:10.1371/journal.pone.0098870)

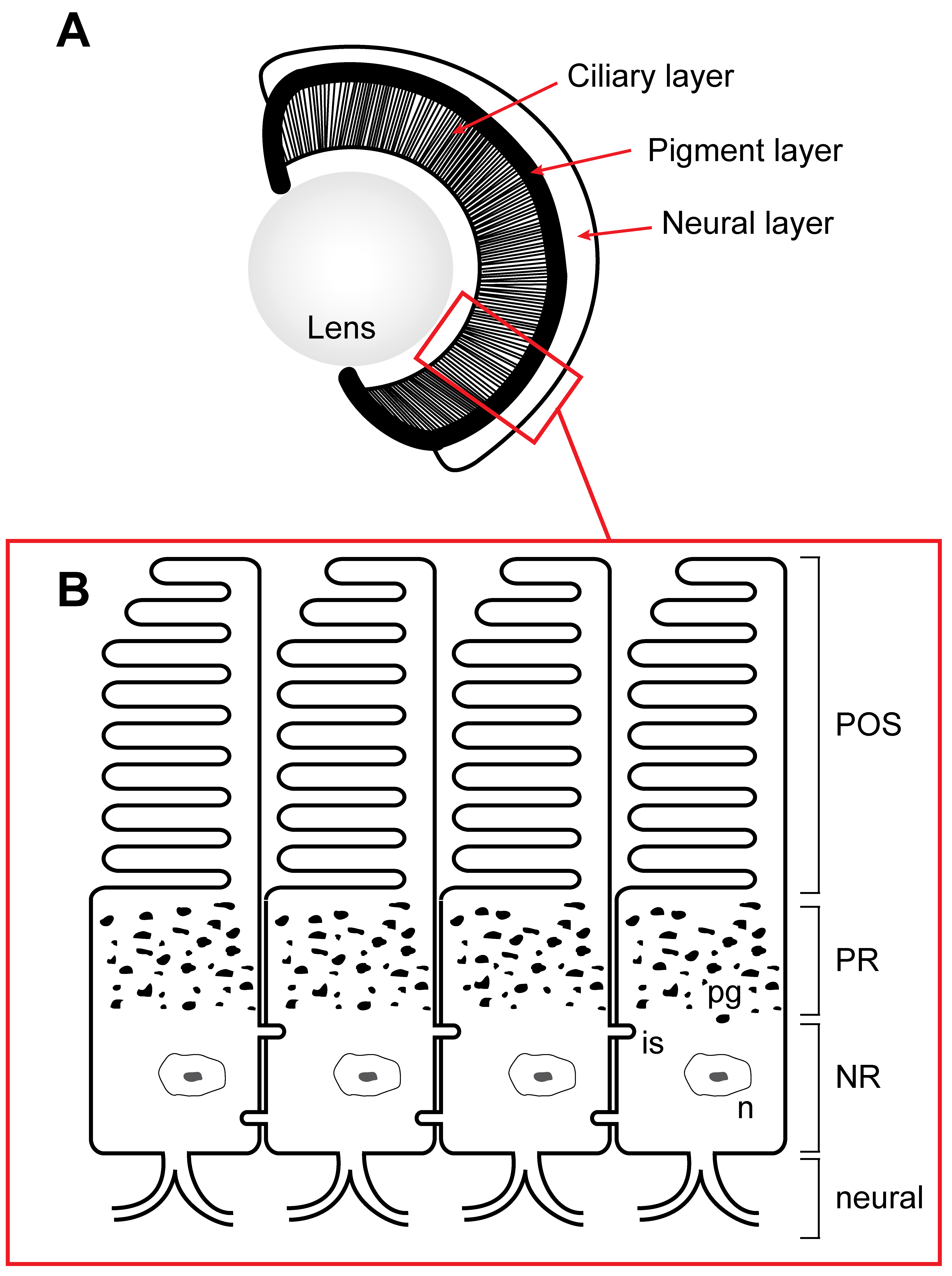

Supplement: Figure S1 — Graphical representation of cubozoan photoreceptor morphology. Sagittal section of the lower lens eye of Tripedalia cystophora (A) (modified from [34]). Light is absorbed in the ciliary layer by the photoreceptive outer segments (POS) (B) and the pigment layer prevents false light entering the eye. Pigment granules (pg) make up the pigment layer and are located in the pigmented region (PR) of the photoreceptors. The nuclei (n) are located in the nuclear region (NR) of the cell bodies. The photoreceptors are everted and the neural layer is thereby located outside the pigment layer. Gray et al. [40] found invaginated synapses (is) in the nuclear region but the significance of this discovery is largely unknown. It is thought that the photoreceptors articulate on second order neurons since the proximal end protrudes into a neural plexus that extends into the neuropil of the rhopalium. (TIF) [file pone.0098870.s001.tif]

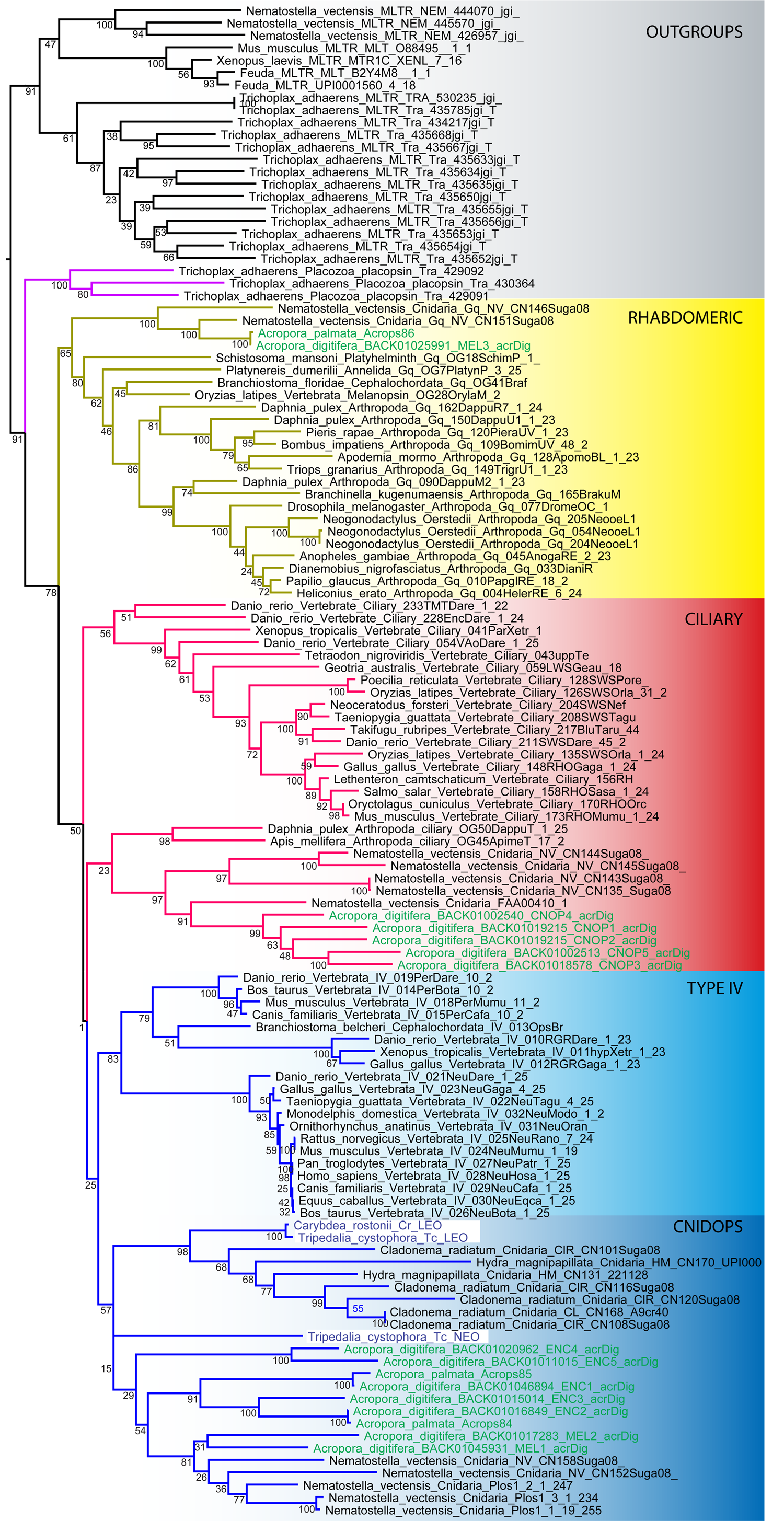

Supplement: Figure S2 — Detailed opsin phylogenetic tree. Maximum likelihood phylogenetic analysis including representative animal opsins from the “O&O” data set of Feuda et al. [27], plus the new Tc-leo gene. Feuda et al. [27] did not include Tc-neo, which we also added to their data set. We rooted animal opsins with melatonin receptor genes (black branches). The branch colors for animal opsins follow Feuda et al. [27]. Unlike Feuda et al. [27], we do not recover monophyletic ciliary opsins (red branches). Also differing from Feuda et al. [27], we do not find a sister-group relationship between ‘cnidops’ [41] genes and the clade called Type IV opsins by Porter et al. [16]. The difference between our topology and that of Feuda et al. [27] seems to be caused by the addition of Tc-neo. Numbers at nodes are bootstrap values based on 100 pseudoreplicated datasets, implemented in RAxML [29], assuming a GTR plus gamma model of protein evolution, the same model used by Feuda et al. [27]. (TIF) [file pone.0098870.s002.tif]
